# Inferior Paralabral Cyst Causing Suspected Axillary Nerve Compression in the Quadrilateral Space: An MRI-Based Case Report

**DOI:** 10.7759/cureus.107361

**Published:** 2026-04-19

**Authors:** Serge M Tzeuton, Joseph Abboud, Eitan Kohan

**Affiliations:** 1 Orthopaedics, University of Maryland Medical Center, Baltimore, USA; 2 Orthopaedic Surgery, Rothman Orthopaedic Institute, Philadelphia, USA; 3 Shoulder and Elbow Surgery, Hackensack University Medical Center, Paramus, USA

**Keywords:** labrum, paralabral cyst, quadilateral space, shoulder, sports

## Abstract

Paralabral cysts are commonly associated with labral tears and can compress nearby neurovascular structures. Inferior paralabral cysts are rare and may extend into the quadrilateral space, placing the axillary nerve at risk. We report a case of a 34-year-old man with severe shoulder pain and minimal objective neurologic deficits in whom MRI demonstrated an anteroinferior labral tear with an associated paralabral cyst extending into the quadrilateral space with suspected compression of the axillary nerve. Although surgical intervention was initially planned, the patient experienced spontaneous resolution of symptoms with conservative management. This case highlights the role of MRI in identifying atypical paralabral cysts and suggests that select patients without significant neurologic deficits may be managed nonoperatively.

## Introduction

Paralabral cysts are frequently associated with labral tears and are most commonly located adjacent to the superior or posterior glenoid, often causing suprascapular nerve compression. Isolated inferior paralabral cysts are rare, occurring as infrequently as 0.6% of all paralabral cysts [1]. It is even less common for these lesions to compress the nearby axillary nerve, a nerve that is most often injured secondary to blunt trauma or traction force [2]. The nerve can be compressed in the quadrilateral space, an area bordered by the teres minor, teres major, long head of the triceps, and surgical neck of the humerus, leading to shoulder pain, lateral deltoid numbness, and, in advanced cases, deltoid weakness or atrophy. Because this occurrence is so rare and clinical findings may be subtle, diagnosis can be challenging. Magnetic resonance imaging (MRI) plays a critical role in identifying both the cyst and its relationship to adjacent neurovascular structures. We report an inferior paralabral cyst with suspected axillary nerve compression that was identified on MRI and successfully managed without surgical intervention. 

## Case presentation

A left-hand dominant 34-year-old man reported a two-year history of intermittent shoulder pain, with acute worsening over the preceding 2-3 months and severe exacerbation five days prior to presentation. The patient reported a VAS 9/10 pain that was located deep within and along the lateral aspect of his shoulder, was worsened with overhead activity, and significantly disrupted his sleep. The patient modified his activity and decreased weightlifting to limit pain. Three days prior to presenting to our clinic, the patient experienced a significant increase in pain that necessitated a visit to the emergency room, where radiographs were unremarkable, and the patient was treated with non-steroidal anti-inflammatories, acetaminophen, and activity modification. He denied any recent specific injury but did recall a shoulder strain from 10 years prior while weightlifting, for which he never sought evaluation or treatment, as the pain resolved on its own. 

On physical examination, his left shoulder demonstrated active forward elevation to 160 degrees, abduction to 50 degrees, external rotation to 30 degrees, and internal rotation to the lateral hip. His left arm had a full passive range of motion with pain at full forward elevation. Neer, Hawkins, and Speed's tests all recreated posterior and lateral shoulder pain. He had negative Kim, Jerk, and O’Brien’s tests. He had no apprehension with abduction and external rotation. He had no muscle atrophy, no fatigability of the deltoid, and 5/5 deltoid and rotator cuff strength. Sensation over the lateral deltoid was intact. He had a normal neurovascular exam distally. His contralateral shoulder exam was unremarkable. 

Given the severity of pain in the absence of objective weakness or neurologic deficits, the underlying etiology was unclear based on examination alone. Initial differential diagnoses included rotator cuff pathology, labral injury, and subacromial impingement. At the first office visit, the patient received a full shoulder radiograph series of the left shoulder, which showed no acute fracture, dislocation, or other pathology. Due to concern for a potential labral tear with associated paralabral cyst and possible nerve compression, an MRI was obtained.

**Figure 1:**
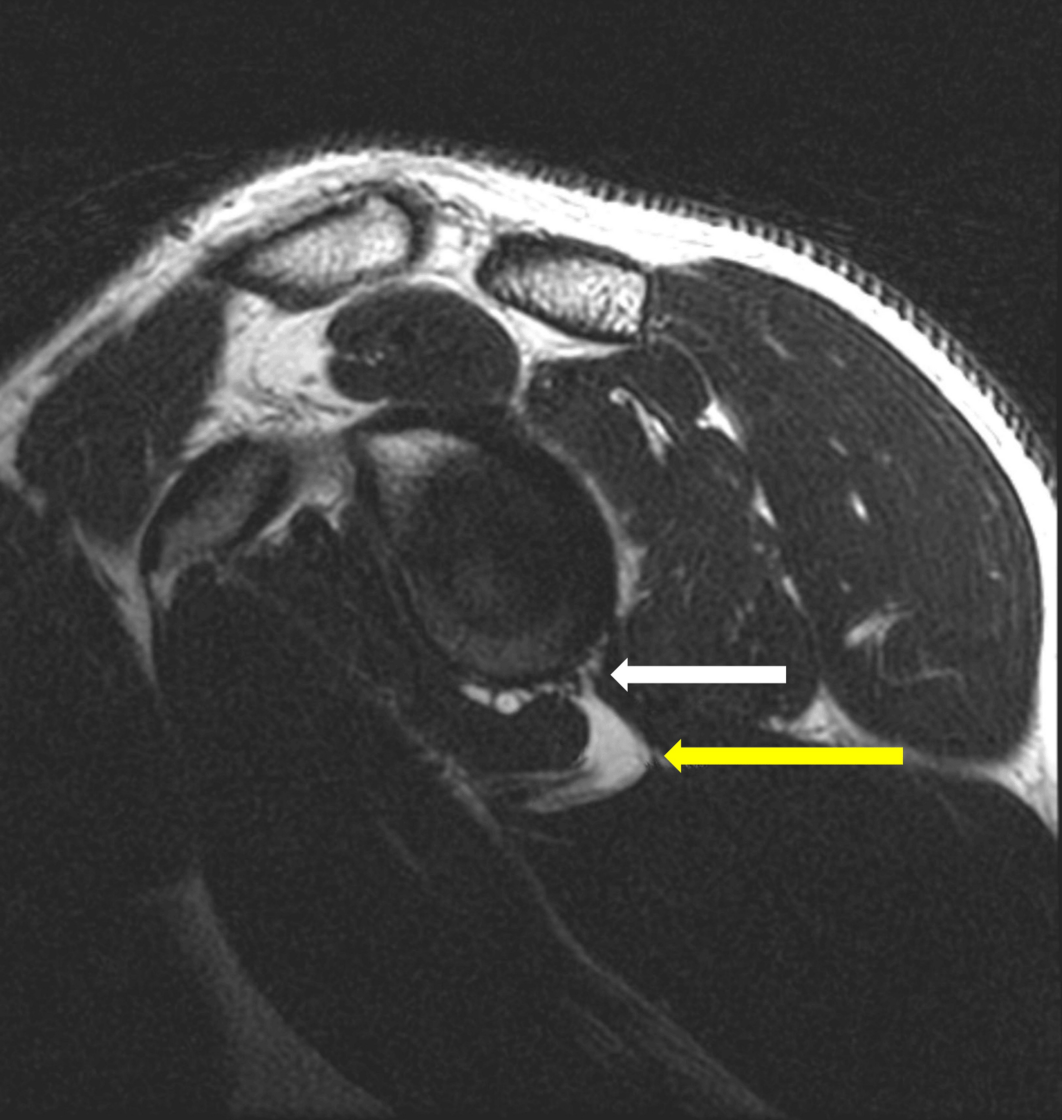
Parasagittal T1-weighted MRI demonstrating a paralabral cyst inferior to the glenoid, extending into the quadrilateral space and abutting the neurovascular bundle The yellow arrow demonstrates the cyst. The white arrow demonstrates the neurovascular bundle

**Figure 2:**
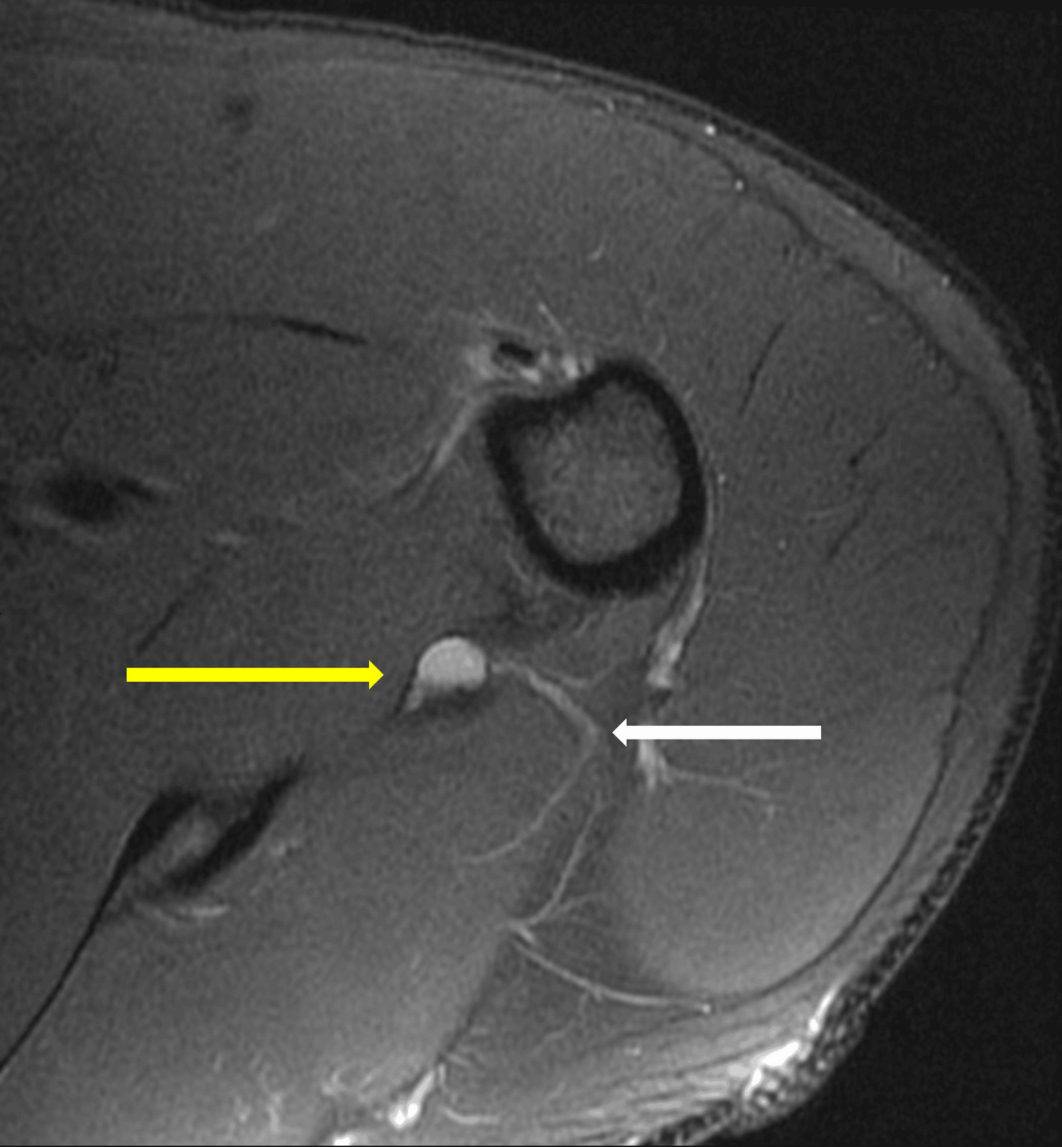
Axial T2 MRI demonstrating the paralabral cyst abutting the axillary nerve within the quadrilateral space. The yellow arrow demonstrates the cyst. The white arrow demonstrates the axillary nerve

The patient’s MRI showed a detached tear of the anterior and inferior labrum with a paralabral cyst along the medial quadrilateral space. The neurovascular bundle was compressed by the cyst on the parasagittal T1 MRI (Figure 1). Axial T2 MRI demonstrated the cyst directly abutting the axillary nerve in the quadrilateral space (Figure 2). Although the radiology report did not identify axillary nerve impingement, the imaging findings raised concern for suspected axillary nerve compression once re-reviewed by the treating team.

Given the severity of pain and concern for possible axillary nerve compression based on MRI findings, surgical intervention was initially planned to decompress the cyst and address the labral pathology. He was instructed to avoid painful movement and lifting of the shoulder, but was not prescribed formal physical therapy. However, at the follow-up visit for MRI review, one week after presentation, the patient reported complete resolution of his pain. Examination demonstrated full active and passive range of motion, with no pain with movement or provocative testing of his shoulder. Given his clinical improvement, the patient was advised to gradually resume activity as tolerated. At subsequent follow-up one week later, the patient continued to be asymptomatic.

## Discussion

Isolated infraclavicular axillary neuropathy is a rare occurrence, with an incidence of 0.3-6.0% of all brachial plexus injuries [2], while paralabral cysts are found in 2-4% of the general population [3]. Inferior paralabral cysts are especially uncommon, comprising as little as 0.6% of all paralabral cysts [1]. The coexistence of these two conditions is rare.

Paralabral cysts are highly correlated with labral tears, and most commonly result in suprascapular nerve compression at the suprascapular or spinoglenoid notch [4-6]. In contrast, inferior paralabral cysts may extend into the quadrilateral space, placing the axillary nerve at risk. Because this is an uncommon location and clinical findings may be subtle, accurate diagnosis can be challenging.

In our patient, severe pain was present without objective weakness, atrophy, or clear neurologic deficits. This highlights the limitations of physical examination alone in localizing pathology. Electrodiagnostic studies were considered but not performed, given rapid symptom resolution. MRI was essential in identifying the anteroinferior labral tear and associated paralabral cyst extending into the quadrilateral space, with suspected compression of the axillary nerve. Notably, the radiology report did not identify nerve impingement, likely due to the subtle findings and the rarity of the condition. This discrepancy highlights the importance of clinician-led image review, particularly in rare presentations where subtle findings may be overlooked.

When patients present with paralabral cysts and only mild symptoms, they may be offered nonoperative treatment such as oral medications and activity modification with reasonable success [7,8]. If symptoms persist or if there are other concerning physical exam findings, such as significant muscle atrophy, surgical intervention is preferred. Surgery most commonly consists of labral repair with cyst decompression [8,9]; however, Schrøder et al. reported that isolated labral repairs without cyst decompression may lead to cyst resolution in some patients [10]. When indicated, surgery is extremely effective at reducing pain and reversing secondary muscle changes (i.e., edema and sometimes, fatty infiltration and atrophy [10]) within months of treatment. 

Only a small number of cases describing axillary nerve compression from inferior paralabral cysts have been reported. Prior reports have typically involved patients with prolonged symptoms, objective weakness, or muscle denervation, and have often been managed surgically [7,11-15]. In contrast, our patient had minimal neurologic findings and experienced rapid symptom resolution with conservative management.

This case highlights two key clinical considerations. First, MRI plays a crucial role in identifying atypically located paralabral cysts and their relationship to adjacent neurovascular structures, particularly when clinical findings are inconclusive. Second, in the absence of objective neurologic deficits, a trial of nonoperative management may be appropriate, as spontaneous resolution of symptoms can occur.

## Conclusions

We report a case of an anteroinferior labral tear with an associated inferior paralabral cyst and suspected axillary nerve compression. Though standard treatment is often surgical labral repair with cyst decompression, clinical judgment should be used in assessing treatment options. In patients presenting with pain alone who report complete pain resolution without intervention, conservative management can be a successful approach. Gradual resumption of normal activity may help prevent recurrence of symptoms.
